# A pilot study comparing the efficacy of autologous cultured fibroblast injections with hyaluronic acid fillers for treating nasolabial folds

**DOI:** 10.1038/s41598-023-33786-9

**Published:** 2023-04-24

**Authors:** Rungsima Wanitphakdeedecha, Janice Natasha C. Ng, Phumithep Phumariyapong, Ya-Nin Nokdhes, Poramin Patthamalai, Ploypailin Tantrapornpong, Panittra Suphatsathienkul, Chalermkwan Apinuntham, Chadakan Yan, Yanisorn Nanchaipruek, Panwadee Thongjaroensirikul, Pitchaya Maneeprasopchoke, Thanya Techapichetvanich, Sasima Eimpunth, Woraphong Manuskiatti, Saowalak Thanachaipiwat, Uraiwan Panich

**Affiliations:** 1 Department of Dermatology, Faculty of Medicine Siriraj Hospital, Mahidol University, 2 Pran-Nok Road, Bangkok Noi 10700, Bangkok, Thailand; 2 Department of Pharmacology, Faculty of Medicine Siriraj Hospital, Mahidol University, Bangkok, Thailand

**Keywords:** Clinical trials, Randomized controlled trials

## Abstract

Autologous cultured fibroblast injections for soft tissue augmentation are a potential alternative to other filler materials. No studies have compared autologous fibroblast injections and hyaluronic acid (HA) fillers for treating nasolabial folds (NLFs). To compare the efficacies and safeties of autologous cultured fibroblast injections and HA fillers for treating NLFs. This prospective, evaluator-blinded, pilot study enrolled 60 Thai female adult patients diagnosed with moderate to severe NLFs. They were randomized to receive either 3 treatments of autologous fibroblasts at 2-week intervals or 1 treatment with HA fillers. The primary outcome was the clinical improvement of the NLFs graded by 2 blinded dermatologists immediately after injection and at 1-, 3-, 6-, and 12-month follow-ups. Objective measurement of the NLF volume was evaluated. Patient self-assessment scores, pain scores, and adverse reactions were recorded. Of the 60 patients, 55 (91.7%) completed the study protocol. The NLF volumes improved significantly in the autologous fibroblast group at all follow-ups relative to baseline (*P* = 0.000, 0.004, 0.000, 0.000, and 0.003). The patients in the autologous fibroblast group rated more noticeable NLF improvements than those in the HA filler group (3-month follow-up, 58.41% vs. 54.67%; 6-month follow-up, 52.50% vs. 46%; 12-month follow-up, 44.55% vs. 31.33%). No serious adverse reactions were recorded. Autologous fibroblast injections are safe and effective for treating NLFs. These injections also promise sustained growth of living cells, possibly leading to a greater persistence than shown by other fillers.

## Introduction

Skin aging is caused by genetic and environmental factors such as sun exposure, air pollution, smoking, alcohol abuse, and poor nutrition^1^. Histologically, aged skin has a thinner epidermis, atrophic dermis, and reduced amounts of subdermal adipose tissue, fibroblasts, and collagen^2^. The most common dermatological presentations of aging are xerosis, skin laxity, wrinkles, and benign lesions^1^.

Dermal fillers are commonly used for skin rejuvenation since they fill up wrinkles and replace soft-tissue volume lost due to aging^3^. Several types of fillers have been approved by the US Food and Drug Administration (US FDA), and they are categorized as temporary, semipermanent, and permanent^4^. Bovine collagen was the first US FDA-approved injectable filler. It has a short-lasting effect and is associated with hypersensitivity reactions^5^. Hyaluronic acid (HA) fillers have become popular. They offer a low immunogenic profile, safety, a range of applications, and reversibility with hyaluronidase^6^. However, severe complications have been reported. They include hypersensitivity reactions, foreign body granuloma, vascular occlusion, skin necrosis, and blindness^7–9^.

Autologous fat transfer (AFT) is another treatment option. It does not produce hypersensitivity reactions and granuloma formation because of the biocompatibility of the adipose tissues used^10^. However, in a retrospective study, 7 patients developed retinal artery occlusion after AFT, and they had a worse final best-corrected visual acuity than other injectables^11^.

In 2011, the US FDA approved Laviv (Azficel-T; Fibrocell Technologies, Inc., Exton, PA, USA) as an autologous fibroblast tissue filler to improve moderate to severe nasolabial folds (NLFs) in adults^12^. Autologous cultured fibroblast injections were safe and effective in improving wrinkles, acne scars, and other dermal defects for up to 12 months after administration with no known side effects^13^. Histologically, fibroblast injections stimulate collagen formation with a concomitant increase in the thickness and density of dermal collagen^13^. Other mechanisms include an induced proliferation of native fibroblasts, secretion of cofactors that can augment the dermis, and the growth of the transplanted fibroblasts^14^.

The objective of this study was to compare the efficacies and safeties of autologous cultured fibroblast injections and HA fillers for treating NLFs.

## Materials and methods

This prospective, single-center, evaluator-blinded pilot study enrolled 60 Thai female adult patients. The women had expressed dissatisfaction with their NLFs, scoring − 1 or − 2 on the Subject Wrinkle Assessment Scale (Table 1). They also had moderate to severe NLF grades, documented using the Evaluator Wrinkle Assessment Scale (Table 2). Patients were excluded if they:

- were pregnant or lactating
- had a history of connective tissue disorder, skin cancers, or related diseases
- had previous autologous fibroblast treatment
- had treatment with fillers, lasers, or energy-based devices during the previous 12 months
- had treatment using transdermal drug delivery, chemical peeling, or topical retinoid during the previous month
- were allergic to collagen, meat, dairy products, gentamycin, amphotericin B, or related products

**Table 1:** Subject Wrinkle Assessment Scale^14^.

How do you feel about the wrinkles in the lower part of your face today?	
Score	Description
− 2	I am very dissatisfied with the wrinkles of the lower part of my face
− 1	I am dissatisfied with the wrinkles of the lower part of my face
0	I am somewhat satisfied with the wrinkles of the lower part of my face
+ 1	I am satisfied with the wrinkles of the lower part of my face
+ 2	I am very satisfied with the wrinkles of the lower part of my face

**Table 2:** Evaluator Wrinkle Severity Assessment Scale^14^.

How do you rate the wrinkles in the lower part of the patient’s face today?	
Score	Description
0	No wrinkles visible
1	Just perceptible wrinkles
2	Shallow wrinkles
3	Moderately deep wrinkles (definite and distinct wrinkles)
4	Deep wrinkles, well-defined edges (prominent wrinkles, well defined edges)
5	Very deep wrinkles, redundant folds (very severe wrinkles, pronounced edges)

The subjects were randomly divided following simple randomization procedures into 2 groups (“HA filler” and “autologous fibroblast”). The HA filler group was given a single injection of HA filler (Restylane; Q-Med AB, Uppsala, Sweden) on both NLFs (0.5 ml on each side) by using 22-guage cannula. The autologous fibroblast group was intradermally injected with autologous cultured fibroblasts on both NLFs (0.5 ml on each side) by using 30-guage needle. However, the fibroblast injections were administered in 3 sessions at 2-week intervals.

### Autologous fibroblast preparation

Preoperatively, each patient from the autologous fibroblast group had tissue collected from the postauricular area. An injection of 2% lidocaine was given, and a 3-mm punch biopsy was performed. The donor site was closed with a single nylon (5.0) suture. The collected tissue was sterilized with povidone-iodine, and the fibroblasts were extracted with debris removal. Separation of epidermis from dermis was done using dispase in fibroblast basal medium at 2–4 °C for 1–4 h. Then, the dermis was cut into small pieces and digested in trypsin at 37 °C for 15 min to isolate dermal fibroblasts. Subsequently, the cells were cultured in fibroblast basal medium with 2% fibroblast growth medium, 1% penicillin (100 units/ml) and streptomycin (100 µg/ml) at 37 °C in a humidified air of 5% CO_2_ (P_CO2_ = 40 Torr). Media was changed every 3 days and the collected fibroblasts were then expanded to 20 × 10^6^ cells per 1 mL of normal saline solution. As observed by light microscopy, the dermal fibroblasts were identified based on their spindle-shaped morphological features. Prior to injection, all specimens underwent standard laboratory testing: sterility using membrane filtration, endotoxin test using Limulus amoebocyte lysate, mycoplasma detection using quantitative polymerase chain reaction, and Gram staining.

### Efficacy and safety assessment

The primary outcome of the study was the clinical improvement of the NLFs. The improvements were graded using a 5-point scale: 2 = “much improved,” 1 = “improved,” 0 = “no change,” − 1 = “worsened,” and − 2 = “much worsened.” Two blinded dermatologists subjectively evaluated photographs at the 1-, 3-, 6-, and 12-month follow-ups. All clinical photographs were taken with identical camera settings, lighting, and positioning using a Canon PowerShot G9 standoff camera (OMNIA Imaging System, Canfield Scientific, Inc., Fairfield, NJ, USA).

In addition, the NLF facial volumes were objectively evaluated using three-dimensional photographs captured by a Vectra H1 Imaging System (Canfield Scientific Inc.) immediately after injection and at the 1-, 3-, 6-, and 12-month follow-ups. Patients also performed self-assessments using the same 5-point scale at each follow-up visit. In addition, the patient pain experienced while the injections were being administered was rated using a 10-point visual analog scale (VAS). Any adverse reactions were recorded.

Statistical analyses were performed using PASW Statistics for Windows, version 18.0 (SPSS Inc., Chicago, IL, USA). Descriptive analysis was used for demographic data. Nasolabial folds volume changes were calculated using repeated measures ANOVA and paired t-test. Subjective improvement evaluation was analyzed by using Wilcoxon signed-ranks test. A probability (*P*) value < 0.05 was considered statistically significant.

The Ethics Committee of the Siriraj Institutional Review Board approved this study (approval number si690/2014). The study was performed per the Helsinki Declaration of 1964 and subsequent amendments. Written informed consent was obtained from all patients before their enrollment.

### Ethics approval

The study was approved by the Ethics Committee of the Siriraj Institutional Review Board (Approval No. SI690/2014). Written informed consent was obtained for the publication and use of images prior to patients’ enrollment in the study. This study was performed in accordance with the Helsinki Declaration of 1964 and its subsequent amendments and was registered at ClinicalTrials.gov (NCT No. TCTR20220217002, 17/02/2022).

## Results

Of the 60 female patients recruited, 55 (91.7%) completed the study protocol and were included in the final analysis. Five patients were withdrawn because they could not attend all follow-up visits. There were 30 patients in the HA group (mean age, 39.45 ± 9.89 years) and 25 in the autologous fibroblast group (mean age, 43.44 ± 8.91 years).

The objective evaluations of the volume differences in the NLFs using the Vectra H1 Imaging System are presented in Table 3. There was a significant volume improvement in the HA filler group immediately after and at the 1-month follow-up compared with baseline (*P* = 0.000 and 0.000, respectively). In contrast, the autologous fibroblast group showed significant volume improvements at all follow-ups compared with baseline (*P* = 0.000, 0.004, 0.000, 0.000, and 0.003). Furthermore, there was a significant difference between the 2 groups immediately after the HA filler injection and the first of the 3 fibroblast injections (*P* = 0.034).

**Table 3:** Evaluation of the volume difference in the nasolabial folds using Vectra H1 Imaging System.

Volume difference (ml)	HA filler Group	P-value^a^	Autologous fibroblast group	P-value^b^	P-value^c^
Immediately after	0.43 ± 0.50	0.000*	0.26 ± 0.31	0.000*	0.034*
1-month follow-up	0.40 ± 0.83	0.000*	0.17 ± 0.41	0.004*	0.070
3-month follow-up	− 0.06 ± 1.41	0.733	0.20 ± 0.31	0.000*	0.161
6-month follow-up	0.03 ± 1.33	0.854	0.21 ± 0.35	0.000*	0.335
12-month follow-up	− 0.34 ± 2.19	0.253	0.17 ± 0.38	0.003*	0.095

*P value < 0.05.

*P*-value^a^—comparing within HA filler group.

*P*-value^b^—comparing within autologous fibroblast group.

*P*-value^c^—comparing between HA filler group and autologous fibroblast group at the same time point.

**Figure 1:**
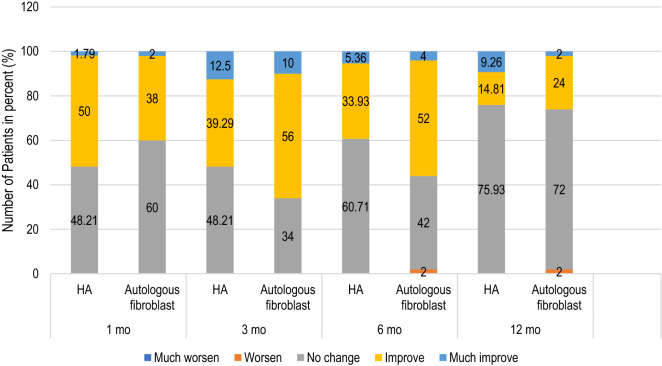
Subjective assessments by blinded dermatologists of the HA and autologous fibroblast groups at all follow-ups.

The subjective evaluations made by the 2 blinded dermatologists of the clinical improvements using the 5-point scale are illustrated in Fig. 1. In the HA group, most patients (50%) showed improvement during the 1-month follow-up compared with baseline. However, at the 3-, 6-, and 12-month follow-ups, most HA patients (48.21%, 60.71%, and 75.93%) were rated with no change. As for the autologous fibroblast group, most (60%) showed no change at the 1-month follow-up compared with baseline. At the 3- and 6-month follow-ups, a slight majority (56% and 52%) showed improvement. At the 12-month follow-up, 72% of the autologous fibroblast group showed no improvement. Inter-rater reliability between evaluators was calculated by using kappa statistics. The correlation coefficient was 0.67 with the *P*-value of 0.00.

**Figure 2:**
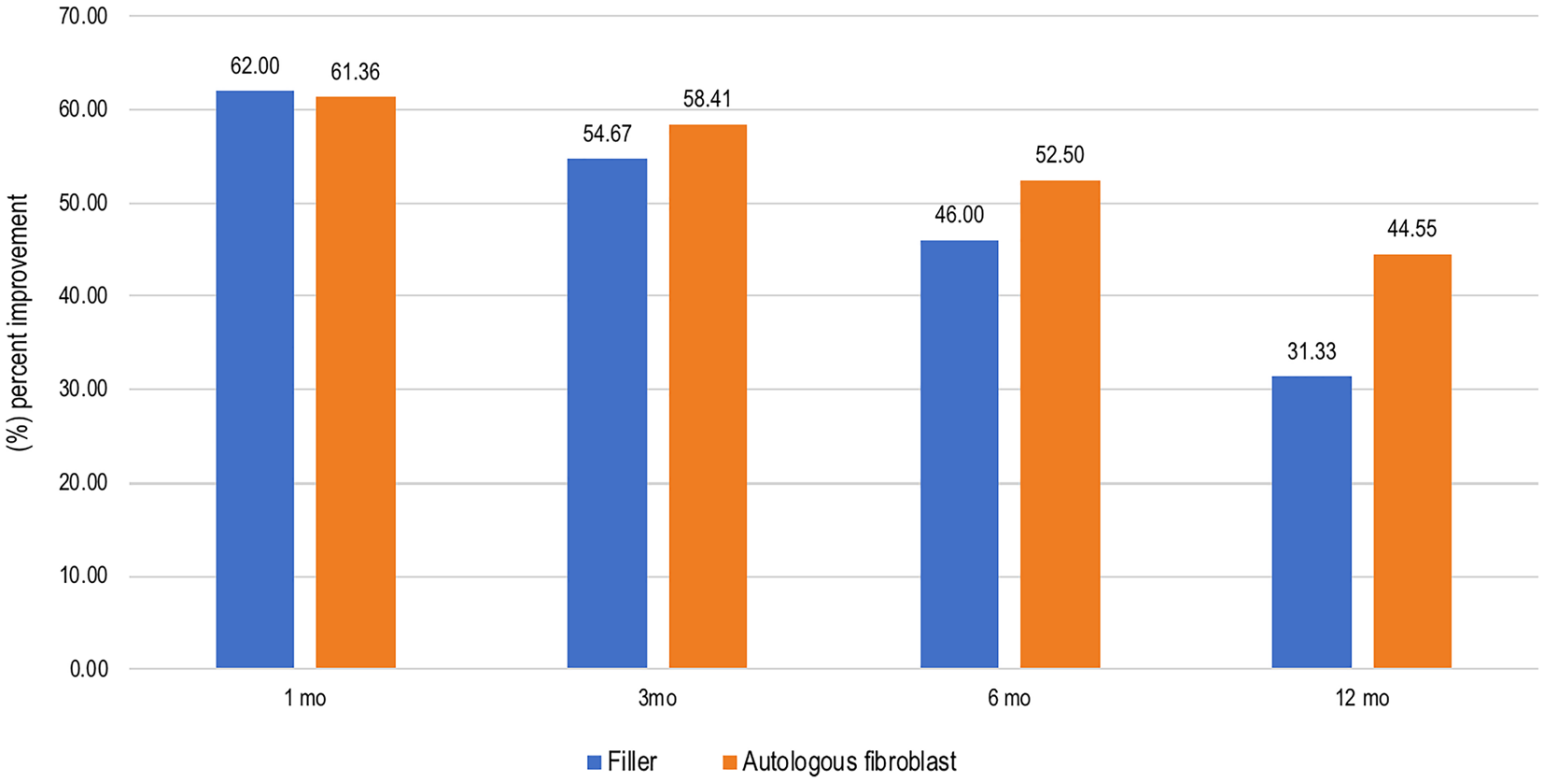
Patient self-assessments of the improvement to NLFs at all follow-ups.

**Figure 3:**
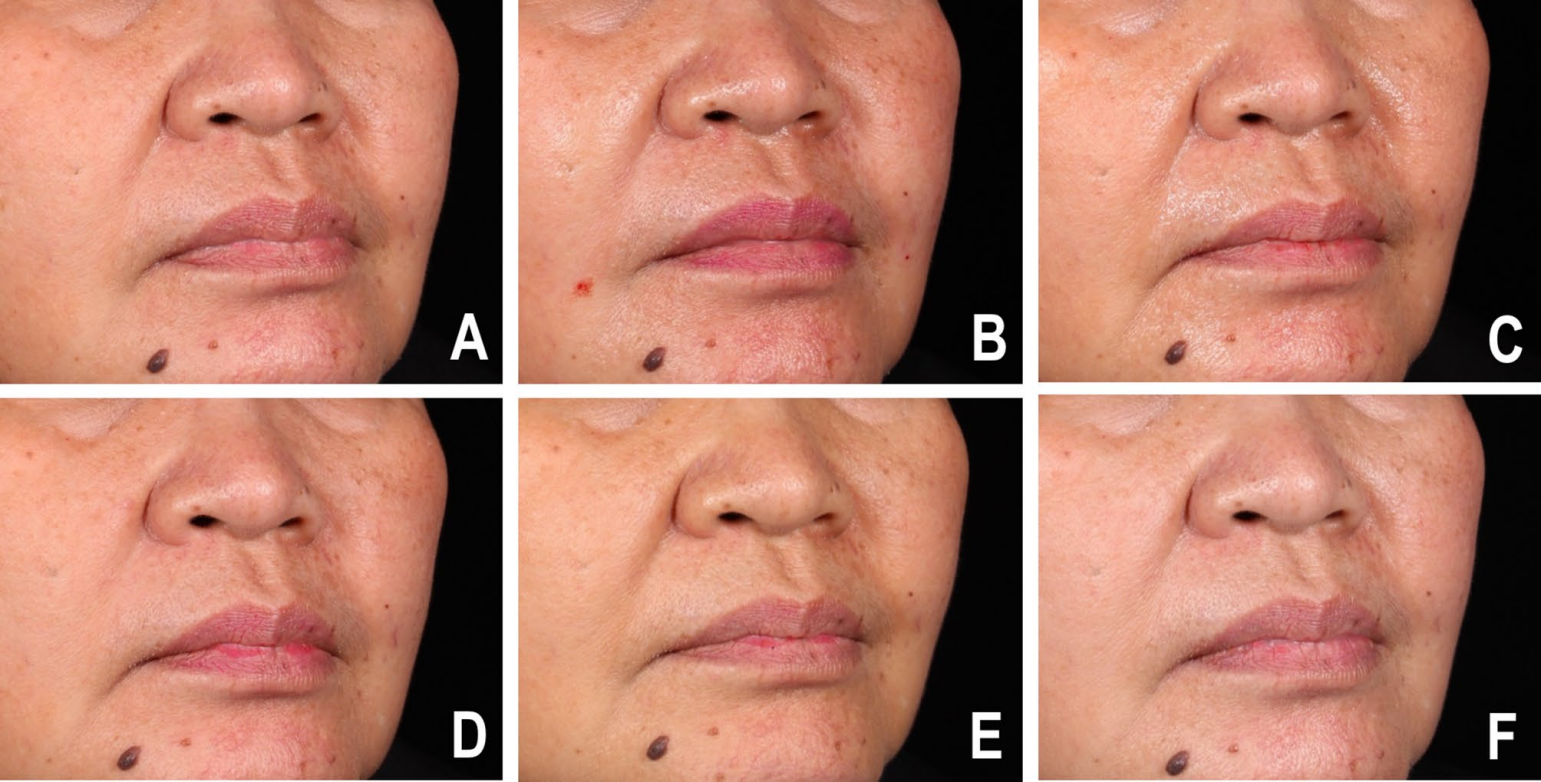
Clinical photographs of an HA filler group participant at (**A**) baseline, (**B**) immediately after injection, (**C**) the 1-month follow-up, (**D**) the 3-month follow-up, (**E**) the 6-month follow-up, and (**F**) the 12-month follow-up.

**Figure 4:**
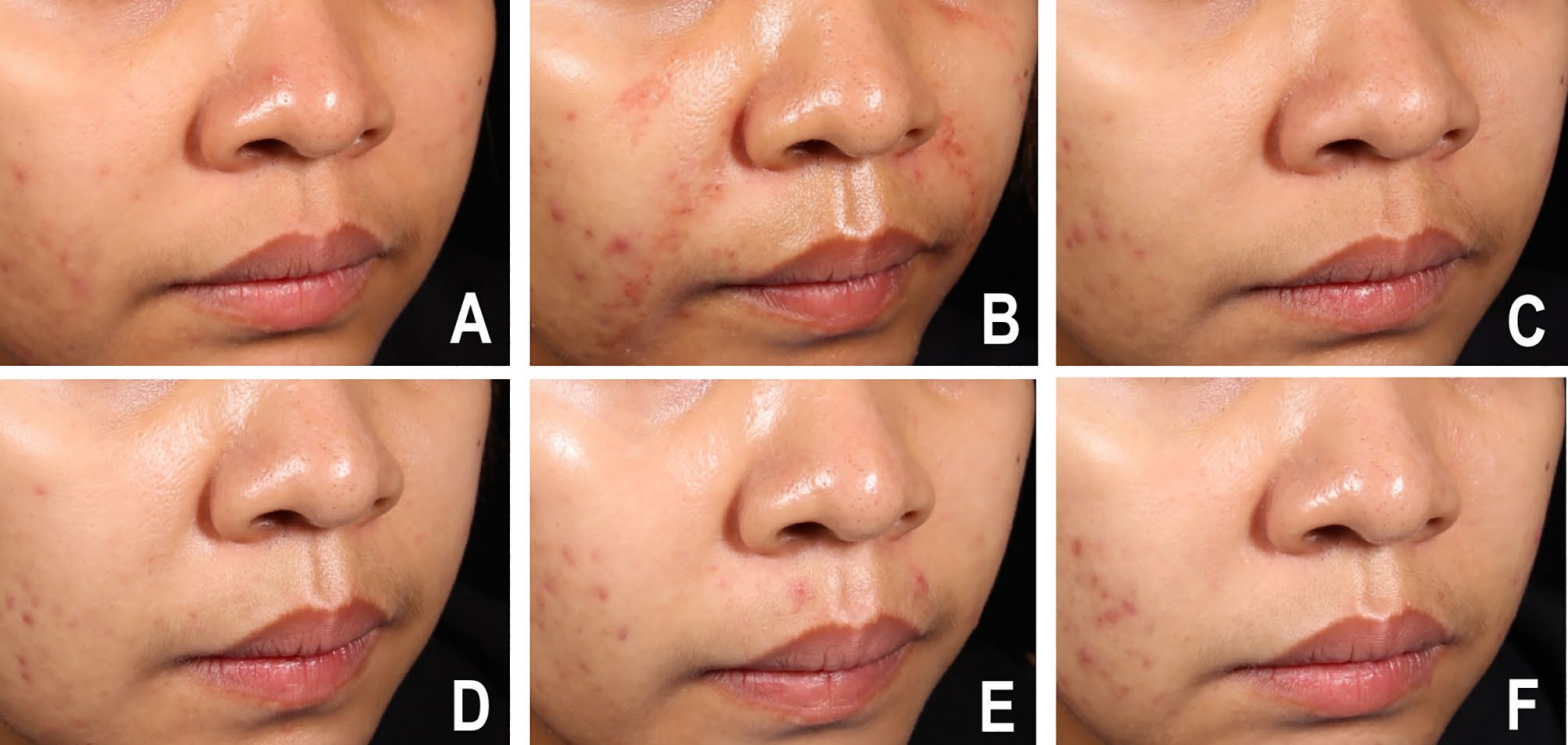
Clinical photographs of an autologous fibroblast group participant at (**A**) baseline, (**B**) immediately after injection, (**C**) the 1-month follow-up, (**D**) the 3-month follow-up, (**E**) the 6-month follow-up, and (**F**) the 12-month follow-up.

Patient self-assessment scores were recorded at the 1-, 3-, 6-, and 12-month follow-ups (Fig. 2). Both treatment groups (62%, 61.36%) reported almost identical values at the 1-month follow-up (62% and 61.36%). At the 3-month follow-up, more patients in the autologous fibroblast group noticed improvement (58.41%) than in the HA group (54.67%). At the 6-month follow-up, 52.50% of the autologous fibroblast group reported improvement compared with 46% of the HA group. At the 12-month follow-up, 44.55% of the autologous fibroblast group said that there had been improvement, compared with 31.33% of the HA group. The clinical improvements to the NLFs after the HA filler and fibroblast injections are depicted in Figs. 3 and 4.

Regarding the patient pain scores, the HA group gave a score of 3.8 out of 10, whereas the autologous fibroblast group rated the injection pain as 5.36 out of 10. As to adverse reactions, 1 patient in the HA group reported a lump at the injection site. It improved after 2 months without any treatment. In the autologous fibroblast group, 1 patient experienced mild transient erythema, but it resolved spontaneously within a day. No serious adverse reactions were found during the study period.

## Discussion

The aging process reduces the number of fibroblasts in the dermis and their ability to synthesize collagen and elastin^15^. Multiple dermal fillers for soft tissue augmentation are available on the market. One type, HA fillers, has become particularly popular in recent years^16^. An ideal filler should have permanence, good biocompatibility, chemical inertness, soft and easy-to-use consistency, and minimal adverse reactions^17^. However, even in the hands of the most experienced physicians, unwanted side effects can occur with dermal fillers. Because autologous fibroblasts offer long-term efficacy and an absence of allergic reactions, they are a natural alternative to filler materials^18^.

In the present study, the HA group demonstrated a significant volume improvement immediately after the HA injection and at the 1-month follow-up compared with baseline (*P* = 0.000 and 0.000, respectively). By contrast, the autologous fibroblast group had significant volume improvements at all follow-ups compared with baseline (*P* = 0.000, 0.004, 0.000, 0.000 and 0.003). These findings prove that using autologous fibroblast injections can produce sustained clinical improvements in skin that has suffered collagen degradation^19^. A previous study biometrically assessed the skin changes caused by autologous fibroblast injections. The research revealed significant increases in epidermal and dermal thicknesses after 6 months relative to the pretreatment values^20^.

In our study, most patients in the HA group had clinical improvements at the 1-month follow-up (50%) compared with baseline. However, no changes were detected at the 3-, 6-, and 12-month follow-ups. The autologous fibroblast group showed improvements only at the 3- and 6-month follow-ups.

Earlier investigations found that HA fillers in NLFs can show clinical results 1 month after injection, with satisfaction maintained at 6 months^21^. In our study, the HA fillers degraded over time, but the autologous fibroblast injections resulted in gradual improvements, as evidenced by our patient self-assessments. Fibroblast injections have previously been shown to require a more extended period before their effects are observed (at least 1–2 months following the completion of treatment) but without any risk of hypersensitivity reactions^13^. The findings of the current investigation are consistent with those of another study that used autologous fibroblasts in NLFs. That research observed sustained soft tissue augmentation at 3 months with continued clinical improvement at 6 months^19^. The more extended period before wrinkle improvement becomes apparent with autologous fibroblast injections than with HA fillers results from collagen deposition not using direct volume replacement. Consequently, fibroblast injections have a more gradual effect than HA fillers, which show immediate results^14^.

More autologous fibroblast patients than HA patients reported improvements in their NLFs at the 3-, 6-, and 12-month follow-ups. This finding is similar to the results of another study. It found that 81.6% of the patients treated with autologous fibroblasts demonstrated continued therapeutic benefits even at the 12-month follow-up^13^.

Other novel autologous fibroblast combinations have been formulated to produce a faster onset of results with more prolonged clinical efficacy. For instance, Jiang et al. combined autologous fibroblasts and keratin as a soft tissue filler. The researchers found that 90% of their patients had significant NLF improvements at the 1-month follow-up. In addition, the improvements were maintained in 93.8% of cases at the 24-month follow-up^22^. In other research, autologous fibroblasts combined with plasma gel (Fibrogel) showed persistent improvements in the infraorbital area and lower face at the 3-, 6-, and 12-month follow-ups, with minimal adverse reactions^23^.

Regarding adverse reactions in the present work, we had 1 patient in the autologous fibroblast group who experienced mild transient erythema. The condition resolved spontaneously within a day. Erythema is a commonly reported adverse reaction among patients injected with autologous fibroblasts^14,20^.

Currently, fibroblast injections are indicated for the treatment of NLFs. Autologous fibroblast treatment of NLFs is a promising option for patients. As autologous fibroblasts are not volume fillers, they are ideal for treating fine lines. On the other hand, possible disadvantages of autologous fibroblasts over HA fillers are their additional costs (which can be as high as 4 times when comparing to 1 cc of HA fillers) and the long lead time needed to harvest and culture the fibroblasts.

Our study was limited by its small sample size, and the follow-up duration might not be long enough to determine the maximum efficacy of the treatment. We recommend that further studies be conducted with larger sample sizes and longer follow-ups to establish the longevity of autologous fibroblast injection therapy.

## Conclusions

Autologous fibroblast injections are safe and effective for treating NLFs. Unlike conventional dermal fillers, autologous cultured fibroblast cells are injected superficially and may require a more extended period to show improvement. They also promise sustained growth of living cells, possibly leading to a greater persistence than shown by other fillers.

## Data Availability

The datasets generated or analyzed during this study are available from the corresponding author on reasonable request.
